# Expression and immune characterization of a novel enzyme, protein arginine methyltransferase 1, from *Schistosoma japonicum*

**DOI:** 10.1007/s00436-013-3723-6

**Published:** 2013-12-17

**Authors:** Wei Diao, Hejun Zhou, Wei Pan, Haipeng Liu, Yujuan Shen, Yuxin Xu, Xiaohong Li, Jianping Cao

**Affiliations:** Key Laboratory of Parasite and Vector Biology, Ministry of Health, WHO Collaborating Center for Malaria, Schistosomiasis and Filariasis, National Institute of Parasitic Diseases, Chinese Center for Disease Control and Prevention, Rui Jin Er Lu 207, Shanghai, 200025 People’s Republic of China

## Abstract

Protein arginine methyltransferase 1 (PRMT1) is an arginine-specific protein methyltransferase that methylates a number of proteins involved in transcription and RNA metabolism in all parasitic helminths, including the human blood fluke, *Schistosoma japonicum*. To characterize the role of PRMT1 in the development of *S. japonicum* and to investigate its influence on parasite–host interactions, we cloned and expressed the protein from an existing cDNA library. We report that the clone encoded a polypeptide comprising 360 amino acids with a predictive Mr of 42 kDa. Bioinformatic analyses predicted that there were many potential B cell epitopes and T cell epitopes associated with SjcPRMT1, suggesting it is a potential candidate molecule for vaccine development. The purified recombinant protein of *S. japonicum* (Chinese strain) (rSjcPRMT1) was found to be immunogenic, eliciting a high antibody titer in mice. Moreover, Western blot analysis revealed that the protein could be recognized by the sera of infected mice. Using flow cytometry, we showed that rSjcPRMT1 slightly upregulated the expression of CD40, CD80, CD86, and MHC-II molecules of mouse bone marrow-derived dendritic cell (BMDC), indicating that rSjcPRMT1 could induce mouse BMDC to mature and, therefore, activate their immune response. Overall, our findings provide evidence that rSjcPRMT1 could serve as an effective candidate molecule for the development of a vaccine against infection with *S. japonicum*.

## Introduction

Schistosomiasis is a widespread infectious disease that constitutes a serious public health problem worldwide. While significant progress has been made in treatment of the disease with safer and more effective drugs now available, treatment does not prevent reinfection (Patz et al. 2000; McManus and Loukas 2008). Moreover, reinfection rates are high, which results in continuing outbreaks of high prevalence and high associated morbidity (McManus 2005). Vaccination offers an effective alternative to the treatment of schistosomiasis. However, to date, vaccines have not been used in clinical trials because their protective efficiency remains low (Xin et al. 2006; Siddiqui et al. 2011). Hence, there is an urgent need to identify and develop candidate molecules for use in future vaccines to protect again schistosomiasis. The reed vole, *Microtus fortis* (herein referred to as Mf), is the only known mammalian host in which schistosomes of *Schistosoma japonicum* are unable to mature, and thus, cause significant pathogenesis in hosts (Liu et al. 2001; Sun et al. 2004; Peng et al. 2011). Therefore, investigating the immunogenetic response of Mf to schistosome infection could help to identify new candidate vaccine molecules that protect this species from the debilitating effects of schistosomiasis.

Previously, we constructed and screened the cDNA library of schistosomu using the serum of wild-type Mf and identified 26 candidate genes likely involved in the immune response of Mf, including protein arginine methyltransferase 1 (PRMT1), high mobility group box-1(HMGB1), cytochrome b5, mitochondrion coding region, 16 *S. japonicum* proteins of unknown function, and six novel encoded proteins (data not shown). Protein arginine methyltransferase 1 (PRMT1) is a type I enzyme that catalyzes the transfer of an S-adenosyl-l-methionine to a broad spectrum of substrates, including histones, RNA-transporting proteins, and nuclear hormone receptor coactivators (Chiou et al. 2007). Protein arginine methylation is also involved in regulating various cellular processes including transcription regulation, DNA repair, RNA processing, and signal transduction (Bedford and Richard 2005; Hung et al. 2004; Krause et al. 2007). Hence, it is likely that this molecule could prove effective in the development of a vaccine against schistosomiasis. However, its role in host immune regulation following infection with *S. japonicum* infection remains elusive. In the present study, we aimed to characterize the role of PRMT1 in the development of *S. japonicum* and its influence on parasite–host interactions. To do so, we cloned and expressed the protein PRMT1 of *S. japonicum* (Chinese strain) (rSjcRPMI1) from our existing cDNA library and investigated its effects on immune responses in this host species.

## Materials and methods

### Ethics statement

This study was carried out in strict accordance with the recommendations in the Guide for the Care and Use of Laboratory Animals of the National Institute of Parasitic Diseases, Chinese Center for Disease Control and Prevention. The protocol was approved by the Laboratory Animal Welfare & Ethics Committee (LAWEC), National Institute of Parasitic Diseases, Chinese Center for Diseases Control and Prevention (permit number: IPD 2011-006). All surgery was performed under sodium pentobarbital anesthesia, and all efforts were made to minimize suffering.

### Amplification and cloning the gene encoding the target protein

RNA was isolated from adult *S. japonicum* (Chinese strain) via digestion, ligation, and sequencing. The gene fragment (SjcRPMI1) was then amplified by reverse transcription PCR (RT-PCR) using the total RNA as a template. The specific primers (sense primer: 5′-GCA GGA TCC ATG AAC GTT AAA AAT GGA GAA GC-3′ and antisense primer: 5′-CGA CTC GAG TCA GCG CAT GCG ATA ATT AAA C-3′, which included a *Bam*H I site and a *Xho* I site, respectively) were designed according to the electronic elongation sequence of SjcPRMT1. The amplification profile used was as follows: 95 °C for 5 min, followed by 35 cycles at 95 °C/45 s, 63 °C/45 s, and 72 °C/1 min 20 s and 72 °C/5 min (final extension). PCR experiments were performed on a programmable Easter-win PCR system using Taq polymerase (Promega, America), and the PCR products were separated on 1.0 % agarose gels and visualized under UV light by ethidium bromide staining. The PCR products were extracted from the agarose gel then double-digested with *Bam*H I and *Xho* I endonucleases and cloned into the prokaryotic expression vector pET28a (Biotec, Beijing, China). Following this the recombinant plasmid, pET28a-SjcPRMT1 was isolated and confirmed by DNA sequencing.

### Prokaryotic expression and purification of the target protein

This plasmid was then transformed into *Escherichia coli* BL21 (DE3) (Biotec, Beijing, China). SjcPRMT1 was expressed as a 6His-SjcPRMT1 (His-SjcPRMT1) fusion protein upon isopropyl-d-thiogalactopyranoside induction (1 mg/mL for 6 h). The recombinant protein was purified by affinity column chromatography using a HISTrap™ FF resin column (GE Healthcare, Milwaukee, WI, USA) followed by thrombin (Novagen) cleavage. Protein samples were fractionated using 15 % sodium dodecyl sulfate polyacrylamide gel electrophoresis (SDS–PAGE) and visualized by Coomassie brilliant blue G-250 staining. The cleaved protein was excised from an SDS–PAGE gel, and its identity was confirmed by mass spectrometry. The protein concentration was determined by the method of Bradford (1976).

### Western blotting analysis

The purified recombinant His-SjcPRMT1 fusion protein was subjected to 15 % SDS–PAGE, electrotransferred to nitrocellulose membrane (Osmonics), and probed with mouse anti-SjcPRMT1 sera (1:1,500 dilution in phosphate-buffered saline [PBS]) as the primary antibody and horseradish peroxidase-conjugated rabbit anti-mouse immunoglobulin G (Sigma) as the secondary antibody. The signal was detected by an electrochemiluminescence Western blotting system (GE Healthcare/Amersham Biosciences). In addition, the purified recombinant SjcPRMT1 was probed with mouse serum (1:500) infected with the cercariae of *S. japonicum*.

### Dendritic cells stimulation and maturation

Dendritic cells (DC) are the most potent antigen-presenting cells and play a major role in the initiation and regulation of the adaptive immune response to antigens. The solution of thrombin-cleaved SjcPRMT1 protein was filtered through a 0.22-μm syringe filter (Acrodisc, Pall, Auckland, USA) and then applied to the Affinity Park™ Detoxi-gel TM Endotoxin Removing Gel column (Pierce, USA) to remove the endotoxin in the protein solution according to the manufacturer's instructions. This allowed the concentrations of SjcPRMT1 and the endotoxin in the elution to be determined. After being cultured for 8 days, the DCs derived from mouse bone marrow were incubated with 0.05, 0.1, 1, or 10 μg/mL of rSjcPRMT1 for 24 h. Lipopolysaccharide (LPS; 0.1 μg/ml) and PBS were used to replace the protein as the positive and negative control, respectively.

### Phenotypic characterization of mouse bone marrow-derived DC after stimulation with PRMT 1

The expression of surface molecules MHC-II and CD11c on collected cells was analyzed using flow cytometry (Calibar BD, USA). To determine the differences of the expression of co-stimulation molecules of DCs, the CD11c^+^ cells were purified using anti-CD11c antibody-conjugated beads (Miltenyi, Germany). They were then analyzed for the expression of CD40, CD80, CD86, and MHC-II.

At each step of the staining, 5 × 10^5^ cells were stained with specific Abs for 30 min at 4 °C in 100 μL of PBS containing 1 % of bovine serum albumin (BSA) and 0.1 % of sodium azide (PBS + BSA + NaN3). We used fluorescein isothiocyanate (FITC) or phycoerythrin (PE)-labeled monoclonal Abs, including PE-conjugated MHC class II (I-Ab, mouse IgG2a, AF6-120.1), FITC-conjugated CD11c (hamster IgG, HL3), PE-conjugated CD40 (hamster IgM, HM40-3), FITC-conjugated CD80 (rat IgG2a, 1G10), and FITC-conjugated CD86 (rat IgG2a, BL1). FITC- or PE-labeled mouse, rat, or hamster IgG was substituted for specific Abs for the negative controls. All Abs were purchased from Peprotech (England).

Gene Runner (Hastings Software, Inc.) was used to predict the secondary structure and the functional sites.

## Results

### Amplification and cloning of the gene encoding SjcPRMT1

**Fig. 1:**
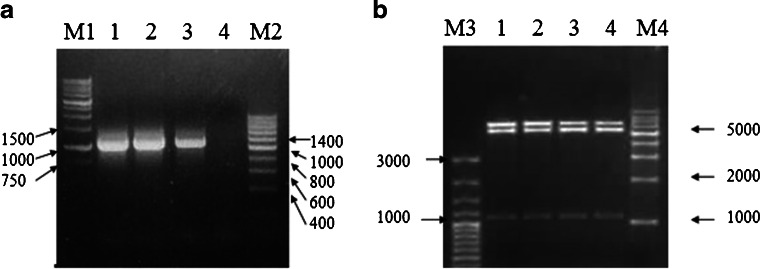
Amplification and cloning of SjcPRMT1. **a** RT–PCR results of SjcPRMT1. The PCR product was approximately 1,100 bp in size. **b** Identification of the recombinant plasmid of pET28a-SjcPRMT1 by double digestion. The recombinant plasmid DNA was digested by *Bam*H I and *Xho* I M1, M2, M3, M4 are all DNA markers

RT–PCR amplification of SjcPRMT1 was successful with a single band revealed by agarose gel electrophoresis of approximately 1,100 bp size (Fig. 1a, lanes 1–3). This PCR product was cloned into the pET-28a vector to generate the pET-28a-SjcPRMT1 recombinant plasmid (confirmed by DNA sequencing and double digestion with *BamH* I and *Xho* I; Fig. 1b, lanes 1–4).

### Bioinformatics analysis of the target protein SjcPRMT1

Using a public analysis software in the website CBS (http://www.cbs.dtu.dk/services/), a bioinformatic analysis of the SjcPRMT1 protein showed that its secondary structure contained numerous α-helix, β-turn, and β-sheet structures, but no transmembrane structure (TM). Prosite motif research, meanwhile, showed that this protein carried seven protein kinase C phosphorylation sites, 3 N-myristoylation sites, 6 casein kinase II phosphorylation sites, 1 Asn-glycosylation site, and 1 cAMP phosphorylation site. In addition, BepiPred 1.0 Server analysis (http://www.cbs.dtu.dk/services/BepiPred/) predicted the presence of many potential B cell epitopes and T cell epitopes in the SjcPRMT1 protein, suggesting it may be a good candidate for vaccine development.

### Expression and Western blotting analysis

**Fig. 2:**
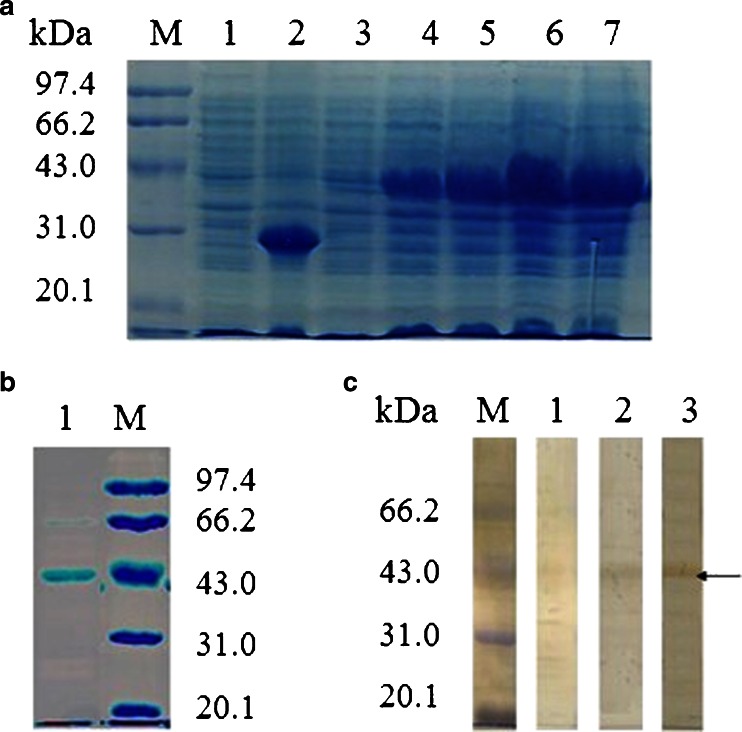
Expression and identification of SjcPRMT1. **a** Analysis of recombinant SjcPRMT1 by SDS–PAGE. *M* protein marker, *1* pET28a/BL21 before isopropyl β-D-1-thiogalactopyranoside (IPTG) inducing, *2* pET28a/BL21 induced by IPTG after 4 h, *3* pET28a-SjcPRMT1/BL21 before IPTG inducing, *4–7* pET28a-SjcPRMT1/BL21 induced by IPTG after 1, 2, 4, and 7 h. **b** SDS–PAGE analysis of purified reSjcPRMT1. **c** Identification of reSjcPRMT1. *M* protein marker, *1* normal mouse serum, *2* anti-His-G HRP antibody, *3* serum from mice infected with *S. japonicum*

The recombinant SjcPRMT1 fusion protein was expressed in *E. coli* BL21 (DE3) and purified by affinity column chromatography. After dialysis, approximately 30 mg of purified protein was obtained from 2,000 mL of culture. The final concentration of the protein was 0.5 mg/mL in PBS. SDS–PAGE showed that the size of the purified fusion recombinant SjcPRMT1 was 42 kDa (Fig. 2a, lanes 4–7; Fig. 2b, lane 1). Results of the Western blotting analysis showed that both the anti-His-G HRP antibody and the sera from mouse infected with cercaria of *S. japonicum* reacted with the purified recombinant SjcPRMT1 protein (Fig. 2c, lanes 2 and 3), showing a band of approximately 42 kDa.

### SjcPRMT1 upregulated the expression of MHC-II, CD40, CD80, and CD86 in mouse BMDC

**Fig. 3:**
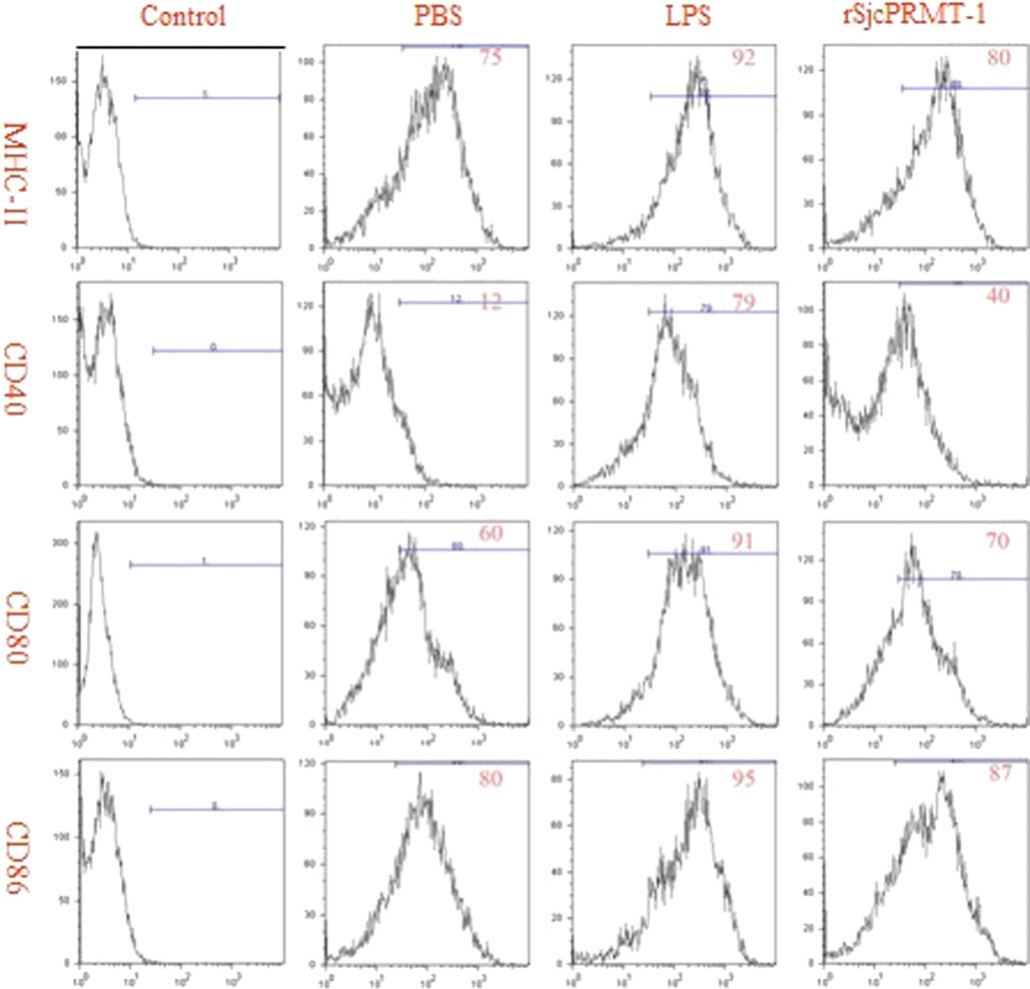
Recombinant SjcPRMT1 regulated expression of surface markers of mouse BMDCs. CD11c^+^ cells were purified using anti-CD11c antibody conjugated beads. The purified DCs with LPS or rSjcPRMT-1 stimulation were stained with indicated mAb and analyzed on an open gate for their phenotype by flow cytometry. *Histograms* represent one of the three flow cytometry experiments

LPS induced DC maturation with upregulation surface expression of MHC-II, CD40, CD80, and CD86 (van der Kleij et al. 2002; Martinez et al. 2007). This was used as the positive stimulation to evaluate the maturation of DCs incubated with SjcPRMT-1. The phenotype of treated DCs was analyzed by flow cytometry. rSjcPRMT-1 significantly activated DCs with upregulation of MHC-II, CD40, CD80, and CD86 (Fig. 3), indicating that rSjcPRMT1 activated DCs and subsequently, T cells.

## Discussion

Vaccination offers an effective alternative to the medical treatment of schistosomiasis, yet remains a poor therapeutic option due to the lack of efficacy of current vaccines (Xin et al. 2006; Siddiqui et al. 2011). Previously, we demonstrated that protein arginine methyltransferase 1 (PRMT1) plays a role in protecting the reed vole, *M. fortis*, from the negative physiological effects of *S. japonicum* infection. In this study, we successfully cloned and expressed the SjRPMI1 protein from our existing cDNA library and found that rSjcPRMI 1 may serve as an effective candidate molecule upon which to base the future development of a vaccine against infection with *S. japonicum*.

Protein arginine methyltransferase 1 (PRMT1), also known as HMT1 hnRNP methyltransferase-like 2, is an arginine-specific protein methyltransferase that methylates a number of proteins involved in transcription and other aspects of RNA metabolism (Xu et al. 2003). It has been shown by three different research groups to be a protein that interacts with TIS21 and BTG1 in a yeast-2-hybrid screen (Lin et al. 1996), to interact with the interferon-alpha receptor (Abramovich et al. 1997), and to be homologous to the yeast homolog Hmt1p (Scott et al. 1998). PRMT1 is not required for adult viability but is required for differentiation during embryogenesis: mice lacking a functional PRMT1 gene die at an early stage of embryogenesis due to failure of the formation of the head and nervous system (Pawlak et al. 2000). The critical nature of PRMT1 in cellular function means that employing antibodies or vaccines to inhibit PRMT1 is likely to result in toxic effects.

In this study, we found that SjcPRMT 1 shared 87 % homology to PRMT1 cloned from the closely related schistosome parasite, *S. mansoni*. This Sm PRMT1 protein has previously been demonstrated to methylate asymmetrically Arg-3 of histone H4 in vitro and to play a role in nuclear receptor-mediated chromatin remodeling and RNA transactions (Mansure et al. 2005). The similarity between Sj PRMT1 and Sm PRMT1 in terms of nucleotide and protein sequences, and the presence of an S-adenosylmethionine binding submotif, suggests that Sj PRMT1 is likely to be the homolog of Sm PRMT1. In addition to cloning and expression of Sj PRMT, we also obtained the purified rSj PRMT 1 protein from *E. coli*. Previous studies have shown that passive transfer of immunized sera among hosts also confers a degree of protection (Moloney et al. 1987); thus, it is assumed that antibodies in the immunized sera protect against *S. japonicum* infection. The result of our antigenicity assay showed that rSjcPRMT1 may be a potential candidate molecule for vaccine development, as it was successfully probed by *S. japonicum* cercariae infected mouse serum. Han et al. (2010) evaluated the protection effects of recombinant PRMT1 against schistosomiasis japonica and found that the protein had a good ability to induce antibodies in mice, which resulted in worm burden reduced by 35.07 % and egg burden reduced by 48.66 %.

PRMT1 gene was found to be expressed in the worm days 7, 13, 18, 23, 32, and 42 (Han et al. 2010) and especially a comparative higher expression in the adult of day 18. In the developmental stage of *S. japonicum*, male–female worm pairing begins at the days 17 to 18, and then the female matured and spawned (Ross et al. 2001). During the period, the nutrition and energy metabolism of worm was especially required for providing related substances to maintain normal growth and eggs production. PRMT1 was therefore speculated to affect the nutrition and energy supplement in the development and maturation course of shistosomula.

Numerous studies have reported that DCs are the most potent antigen-presenting cells in the mammalian body and play a major role in the initiation and regulation of the adaptive immune response to pathogens [Martinez et al. 2007]. The function of these cells in initiating immunity is largely dependent on the expression of MHC-II, CD40, CD80, and CD86 on the surface of DCs themselves (Steinman 1991; Cella et al. 1997; Banchereau and Steinman 1998; Straw et al. 2003). In vivo, only mature DCs with high expression of these molecules can initiate immunity (Lutz and Schuler 2002). We have shown that rSjcPRMT 1 induces DCs to mature via the upregulation of MHC-II, CD40, CD80, and CD86 on DCs surface, which would initiate immunity in vivo. This property makes rSj PRMT 1 a potential antigen candidate, or adjuvant candidate, in the development of a new vaccine. Hence, the results of this study demonstrate that rSjcPRMT1 could induce mouse bone marrow-derived dendritic cell (BMDC) to mature and therefore activate host immune response. We suggest, therefore, that rSjcPRMI 1 may serve as an effective candidate molecule for the development of a vaccine against infection with *S. japonicum*.
